# Idiopathic Subglottic Tracheal Stenosis in Identical Twin Sisters

**Published:** 2019-07

**Authors:** Azizollah Abbasi Dezfouli, Mohammad Behgam Shadmehr, Kambiz Sheikhy

**Affiliations:** 1 *Lung Transplantation Research Center, National Research Institute of Tuberculosis and Lung Diseases (NRITLD), Shahid Beheshti University of Medical Sciences, Tehran, Iran.*; 2 ^*2*^ *Tracheal Diseases Research Center (TDRC), National Research Institute of Tuberculosis and Lung Diseases (NRITLD), Shahid Beheshti University of Medical Sciences, Tehran, Iran.*

**Keywords:** Genetic, Idiopathic, Tracheal stenosis

## Abstract

**Introduction:**

Idiopathic subglottic tracheal stenosis is a rare inflammatory disease of the trachea; most commonly affects females within the age range of 20-50 years. No etiologic factor has yet been identified for this rare tracheal disease and therefore it should be diagnosed after the exclusion of other inflammatory, traumatic, and autoimmune diseases of the trachea. The familial or genetic predisposition to this disease is still unknown although one published report in the literature showed some familial predisposition.

**Case Report:**

A 41-year old woman presented with progressive dyspnea and stridor. The bronchoscopic evaluation revealed subglottic tracheal stenosis; however, there was no significant etiology of this disease after complete evaluations. Therefore, the idiopathic subglottic stenosis was the final diagnosis. After two years, her identical twin sister presented with the same signs and symptoms. There was also no etiology for her tracheal stenosis. The first patient was managed surgically through cricotracheal resection. However, the second sister didn’t need surgical resection due to the mild to moderate tracheal stenosis.

**Conclusion:**

The obtained results of our cases along with the previously reported family cases can potentiate the hypothesis that there is some genetic predisposition to the development of this disease.

## Introduction

Idiopathic subglottic tracheal stenosis is a rare tracheal disease. The first three patients were described by Brandenburg in 1972 (1). The etiologic factor for this type of tracheal stenosis is still unknown, although some authors believe that gastroesophageal reflux disease (GERD) may have a role in the etiology of this disease (2). Moreover, some researchers believe that estrogen can play a role in the etiology of the disease since the disease has a high female preponderance (3). This disease is diagnosed after the exclusion of subglottic stenosis etiologies, such as trauma, prolonged intubation, inflammatory diseases, autoimmune diseases (e.g., granulomatosis with polyangiitis), and tuberculosis. To the best of our knowledge, there is only one report in the literature (4) that suggests a familial predisposition to this disease. In this case report, we reported a pair of adult identical twin sisters who were diagnosed with idiopathic subglottic stenosis, and subjected to its necessary therapeutic procedures. 

## Case Report

Case 1: A 41- year old women presented in 2014 with progressive dyspnea and stridor for two years prior to admission. A rigid bronchoscopy showed normal vocal cords anatomy. However, the circumferential fibrotic stenosis was about 15 mm beneath the vocal cords without any obvious inflammation or granulation tissue, which obliterated about 70% of the airway lumen. The length of the stenosis was about 30 mm and the distal trachea was completely normal. No bronchoscopic dilatation was performed in the management of stenosis. Tissue biopsy revealed mild inflammation and fibrosis with no granulomatous inflammation. There was no history of endotracheal intubation and GERD. Complete laboratory workups for autoimmune diseases, including antineutrophil cytoplasmic antibody (ANCA) and antinuclear antibody, were negative. Bronchoalveolar lavage was negative for Mycobacterium tuberculosis. Based on the severity of stenosis and diagnosis of idiopathic subglottic stenosis, surgical cricotracheal resection was planned for the patient. Anterior part of the cricoid cartilage and the first four rings of the trachea were resected followed by primary laryngotracheal anastomosis. The postoperative course was uneventful and the patient was perfectly asymptomatic after a two-year follow-up. However, the fiberoptic bronchoscopy revealed a small rim of fibrosis at the site of anastomosis with completely patent airway lumen.

**Fig 1:**
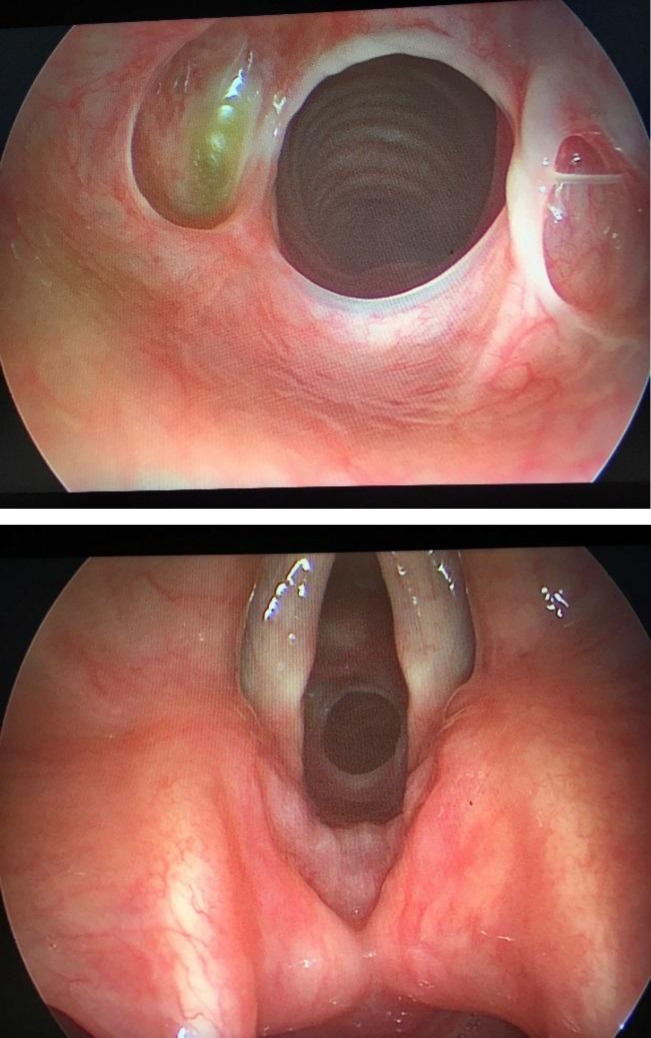
Bronchoscopic view of the subglottic stenosis (A&B)

Case 2: The identical twin sister of the case 1 presented in 2016 with the mild shortness of breath and mild stridor for about 6 months. Again in this patient, past medical history was negative for endotracheal intubation and there was also no history of GERD. All laboratory tests were negative for autoimmune diseases. Bronchoscopy revealed web-like stenosis of about 15 mm distal to vocal cords, which obliterated about 50% of the airway lumen (Figures 1A & 1B). Biopsy confirmed no specific pathology. For this patient, no intervention was performed due to the bronchoscopy and pathological findings and mild symptoms and signs. She is currently doing well and still has mild dyspnea not disturbing her daily activities.

## Discussion

Idiopathic subglottic stenosis is a rare disease. The exact incidence of this condition is unknown and most cases are females within the age range of 20-50 years (3). The etiology of this stenosis is still unknown. Although some researchers believe that estrogen has a role in the pathogenesis of the disease, most studies failed to show any evidence of estrogen receptors at the site of stenosis (3,4). Damrose in his report (3) hypothesized that stenosis at the cricoid or first tracheal ring is caused by durable and severe coughing secondary to the prolonged duration of upper respiratory tract infection. This issue facilitates the frequent telescoping of the first ring to the cricoid cartilage, which may lead to blood supply disruption, and consequently subglottic stenosis (3). Another theory for the etiology of the disease is GERD. A number of patients with idiopathic subglottic stenosis have some evidence of GERD and improved to some extent following the treatment with anti-reflux agents. However, it is not a general finding in all patients with idiopathic subglottic stenosis. In our cases, none of the cases had any symptoms of GERD.

Post-intubation tracheal stenosis, surgery and radiation, infections (e.g., tuberculosis), a series of autoimmune diseases (e.g., granulomatosis with polyangiitis [formerly known as Wegener’s granulomatosis]), and congenital strictures are other causes of subglottic stenosis. It seems that in total about 5% of cases are idiopathic (3), as it was in our two patients. The treatment of idiopathic subglottic stenosis is challenging. Some authors have reported satisfactory results after endoscopic dilation or laser ablation (5). However, most authors believe that the best modality for the treatment is cricotracheal resection and anastomosis (6,7). 

Except in cases with congenital subglottic stenosis that may show some genetic and familial predisposition, there are very limited reports of idiopathic stenosis with familial predisposition. To the best of our knowledge, there is only one report by Dumolin et al. in 2013, which demonstrated this disease in three families. In two families, two sisters were diagnosed with idiopathic subglottic stenosis, and in another family, a mother and her daughter suffered from this disorder. 

## Conclusion

In this case repot, a pair of identical twin sisters were diagnosed with idiopathic subglottic stenosis. Our cases along with the previously reported family cases can potentiate the hypothesis that that there is genetic predisposition to the development of this disease. These findings could lead to more studies on the establishment of the main etiology of this rare tracheal disease.
